# Application of Barophoresis in Chronic Generalized Periodontitis: a Mathematical Substantiation

**DOI:** 10.17691/stm2022.14.4.05

**Published:** 2022-07-29

**Authors:** I.I. Antonov, A.B. Dymnikov, A.A. Muraev, L.A. Ananyeva, S.Yu. Ivanov

**Affiliations:** PhD Student, Department of Maxillofacial Surgery and Surgical Dentistry; Peoples’ Friendship University of Russia, 6 Miklukho-Maklaya St., Moscow, 117198, Russia;; Associate Professor, Department of Maxillofacial Surgery and Surgical Dentistry; Peoples’ Friendship University of Russia, 6 Miklukho-Maklaya St., Moscow, 117198, Russia;; Professor, Department of Maxillofacial Surgery and Surgical Dentistry; Peoples’ Friendship University of Russia, 6 Miklukho-Maklaya St., Moscow, 117198, Russia;; PhD Student, Department of Periodontology; A.I. Yevdokimov Moscow State University of Medicine and Dentistry, 20/1 Delegatskaya St., Moscow, 127473, Russia; ;; Professor, Corresponding Member of the Russian Academy of Sciences, Head of the Department of Maxillofacial Surgery and Surgical Dentistry; Peoples’ Friendship University of Russia, 6 Miklukho-Maklaya St., Moscow, 117198, Russia; Head of the Department of Maxillofacial Surgery; I.M. Sechenov First Moscow State Medical University (Sechenov University), 8/2 Malaya Trubetskaya St., Moscow, 119991, Russia

**Keywords:** barophoresis, periodontitis, periodontology

## Abstract

**The aim of the study** was to evaluate the use of barophoresis for the delivery of liquid-air drug substances to the gums using a mathematical model of the interaction of the drug mixture with periodontal tissues.

**Materials and Methods:**

The solution to the problem was preceded by generation of a geometric CAD model of the device and nozzle for barophoresis, including the nozzle and injector geometry. The Ansys SpaceClaim software package was used to generate the CAD geometry.

**Results:**

When solving the problem of finding the optimal distance from the nozzle to the gum surface, the numerical modeling showed that at a distance of 5 mm, the volume fraction of liquid in the mixture is 18–20%. The mixture actually breaks through the gum, filling 0.8 mm of the gum thickness and spreading symmetrically to the sides at a distance of up to 3 cm, forming a cavity. At a distance of 10 mm from the nozzle to the gum surface, the liquid volume fraction in the mixture close to the gum lies in a narrow range of values of 5 to 7%. The mixture touches the surface of the gums, penetrating slightly — at a distance of 0.30–0.45 mm. At a distance of 15 mm from the nozzle to the gum surface, the volume fraction of liquid in the mixture near the gum lies in the range of 2–5%. The mixture slightly touches the gum surface, getting inside at a distance of up to 0.2 mm, having practically no effect on the gum.

**Conclusion:**

The developed mathematical model confirmed the feasibility of application of barophoresis in the treatment of chronic generalized periodontitis. The optimal distance from the nozzle to the surface should be considered to be 10–15 mm. This distance is safe and allows the drug delivery to a depth of 0.45 mm.

## Introduction

Periodontitis is a multifactorial disease of the musculoskeletal system of the tooth caused by a bacterial infection. According to the WHO, its prevalence reaches 98%. There are different forms of manifestation of periodontitis: an acute localized form is rare, a generalized chronic process as interleaving the phases of exacerbation and remission is more common.

The sign of destructive changes caused by the inflammatory process is the resorption of the bone tissue of the interdental septa and, as a result, the formation of periosteal or intraosseous pathological pockets, depending on the predominant type of bone tissue resorption — either horizontal or vertical.

The periodontal pocket is a space limited by the surface of the tooth root and the alveolar septum, which is filled with subgingival tartar and infected cement attached on the side of the root, as well as granulation immature connective tissue that delimits the bone tissue from the microbial agent. The initiation of the inflammatory process and resorption of bone tissue is caused by periodontopathogens located in the subgingival tooth pocket.

The clinical signs of exacerbation of chronic periodontitis include bleeding and swelling of the gums, suppuration, the presence of supra- and subgingival dental deposits, loss of periodontal attachment, teeth become loose.

The treatment of periodontitis depends on the severity and includes three main stages: conservative therapy; surgery; dynamic follow-up and supportive care. The goal of treatment is to transfer the inflammation from the stage of exacerbation to the stage of long-term controlled remission and reduce the risks of further loss of supporting tissues so as to prevent the development of the risk for tooth loss.

The most common option for conservative treatment of periodontitis is closed curettage of periodontal pockets. It is aimed at the maximum reduction of the concentration of periodontopathogenic microflora by the mechanical removal of subgingival dental plaque using Gracie curettes and ultrasonic tips.

Systemic and local administration of drugs is an important method of drug delivery. In the last half century, the use of systemic administration in the treatment of oral infections has shown some positive effect. However, systemic drug delivery can lead to problems such as dysbiosis and poor biodistribution. Moreover, high doses are usually administered to achieve and maintain effective drug concentrations, which can lead to the development of drug toxicity, gastrointestinal intolerance, and drug resistance. Because of these obvious disadvantages of systemic administration, it is imperative to use local drug delivery systems to place the drugs directly in the periodontal pocket to improve the prevention and treatment of periodontitis. This provides high concentration of active drugs for a sufficiently long time. Local drug delivery vehicles exercise their therapeutic effect mainly through the introduction of three types of drugs: antibacterial drugs (fibers, strips and films, microspheres, nanosystems, gels); inflammation modulators (films, nanosystems, gels); agents that restore alveolar bone and tissue for the treatment of periodontitis (membranes, scaffolds, microspheres, gels, nanosystems) [1].

Recently, a non-contact apparatus technique, barophoresis, has become widely used. The system works with compressed gas, air, any liquid medium, or their mixture.

Barophoresis was first used in dermatology. In particular, the JetPeel transdermal delivery system (5–20-μm microdroplets, pressure of 7 atm, speed of 200 m/s) was tested for topical application in the treatment of androgenetic alopecia [2]. The scalp areas were treated once a week for three months. The composition of the acting aerosol included minoxidil, Mr. Care Hair Vital Ampoule and Mr. Care Hair Vital Ampoule plus. The authors have obtained preliminary positive effects that require further research.

Barophoresis has been widely used in cosmetology for dermabrasion, removal of age spots, smoothing fine wrinkles, skin cleansing, squeezing enlarged pores, hydration and oxygenation of the dermis and epidermis, improving the rheology of microcirculation, dermal lymphatic drainage, as well as for transdermal drug delivery by jet injection [3].

We have not found out any data on the use of barophoresis in dentistry. Obviously, the modes of exposure of the liquid-air mixture on the skin should differ from exposure on the mucous membrane due to the different structure and thickness of the corneous layer. Therefore, before applying the barophoresis system on the mucous membrane, it is necessary to determine the conditions and consequences of the exposure of the liquid-air mixture on it.

**The aim of the study** was to evaluate the possibility of using barophoresis for the delivery of liquid-air drugs to the gums using a mathematical model of the interaction of the drug mixture with periodontal tissues.

## Materials and Methods

The solution to the problem was preceded by generation of a geometric CAD model of the device and nozzle for barophoresis, including the geometry of the nozzle and injectors. The Ansys SpaceClaim software package was used to generate the CAD geometry.

### Principle of operation of the device

The device adapted to a dental unit (a nozzle with a drip system + 3-m long hoses) for barophoresis is operated as follows. A mixture of water-soluble liquid and oxygen is injected into three open converging–expanding Venturi channels, which accelerate the drops to a speed of 200 m/s at the output, thereby creating a powerful microdroplet liquid-air flow with a diameter of 5 to 20 μm, depending on the distance of 5 to 15 mm between the nozzle and the gum. The spray output generated by the compressed air source is supplied at a pressure of 7 atm and mixed with the liquid in the confuser output. The device is operated with a foot pedal switch.

**Figure 1.**
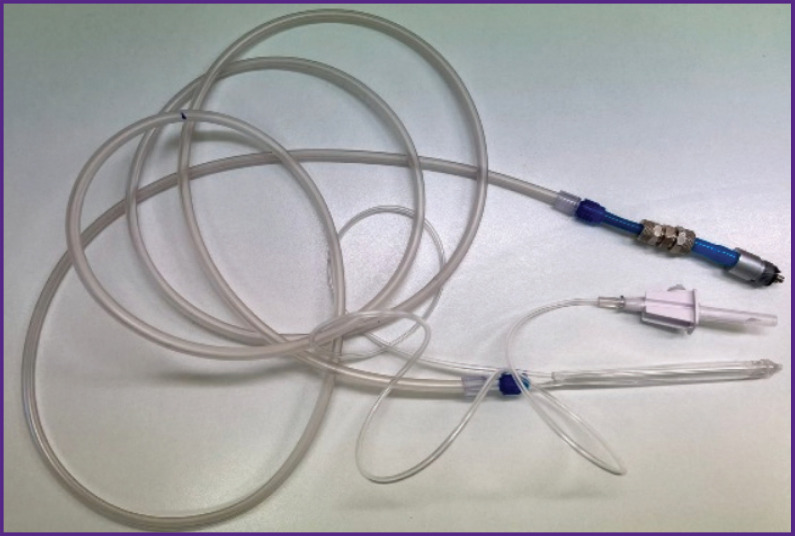
General view of the device for barophoresis

**Figure 2.**
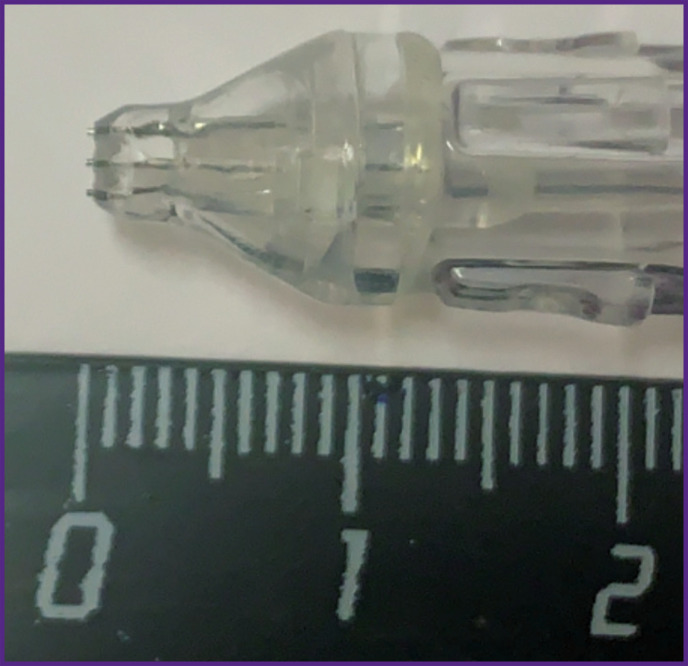
Shape and size of the confuser output

The general view of the device is shown in Figure 1. The nozzle is 15 cm long, the step between the Venturi channels is 1 mm, the diameter is 0.8 mm, the diameter of the air channel is 5 mm. The confuser output for the concentration of the air flow is shown in Figure 2.

### 3D model of a nozzle for barophoresis and the effect of a liquid-air mixture on the gum

**Figure 3.**
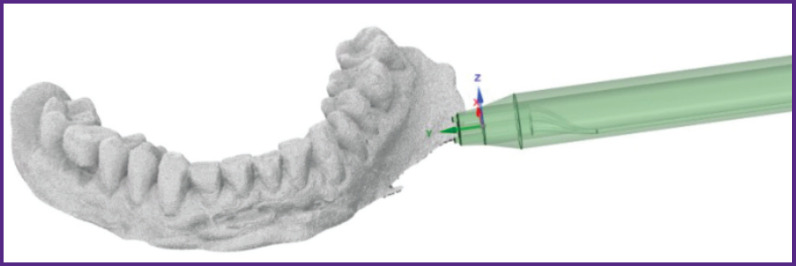
3D model of the nozzle and lower jaw

To consider a mathematical model of the process of the liquid-air mixture formation in the confuser output of the nozzle and calculate the effect of this mixture on the gum, the three-dimensional models of the nozzle and actual-size jaw were created (Figure 3). The model of the nozzle was created in the Ansys SpaceClaim software, the model of the jaw with the gum was obtained by optical scanning of the dentition and the alveolar part of the lower jaw in the oral cavity using the 3Shape 3D scanner (Denmark).

**Figure 4.**
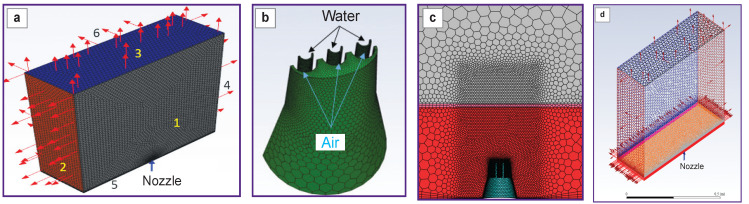
Control-volume computational grid: (a) general view; (b) boundaries with air flow *V*G and liquid *V*L; (c) computational grid in the symmetry plane; (d) general view of the calculated unstructured polyhedral region; *1* — symmetry boundary; *2*, *3*, *4*, *6* — boundaries with pressure *P*=0 Pa; *5* — no-slip impenetrable

The polyhedral unstructured control-volume computational grid (Figure 4) was generated on the basis of the CAD geometry of the nozzle and consisted of ~2 million control volumes; the elements were clustered in the blowing areas. A prismatic layer of 15 layers with a relative growth factor for the correct description of the boundary layer based on the requirements of the *k–ω SST* turbulence model was formed on the boundary surfaces of the wall-type domain.

The air outflow velocity was assumed to be *V*_G_=300 m/s, and that of the liquid was *V*_L_=7 m/s. The volume flow rate of the liquid was 0.00035 m^3^/s, ~0.22 L/min, the total flow rate of the liquid + air mixture was 2.67 kg/s.

The problem was considered in full scale (1:1) in a steady state approach, using the assumption of the symmetry boundary in the pressure-based solver of the Ansys Fluent software.

Since two phases — a liquid with drops of a constant diameter *d*=1·10^–5^ m and air were supposed to be used, a multiphase model of a homogeneous mixture was applied. Heat and mass transfer were not taken into account. The air density, with due regard to the high flow velocity, was taken as the density of an ideal gas with viscosity according to the Sutherland model, the properties of the liquid were considered standard. The calculation was carried out for standard conditions: *P*=10^5^ Pa and *T*=293.15 K.

The Reynolds-averaged Navier–Stokes equations closed by the *k–ω SST* turbulence model were solved iteratively until convergence criteria, which occurred after approx. 256 steps. Both temporal and spatial discretization of control volumes were provided by calculation schemes of the second-order accuracy.

**Figure 5.**
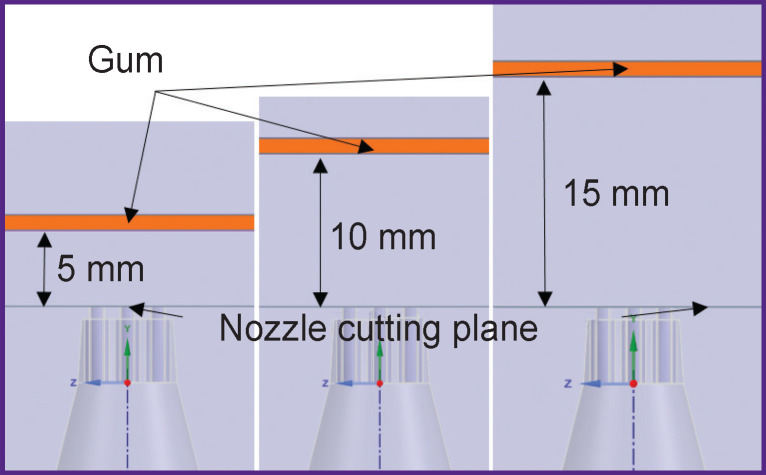
Estimated cases of the mutual position of the nozzle and gum

To determine the penetration depth of the mixture into the gum at different distances of the nozzle from the gum surface (from 5 to 15 mm), three three-dimensional identical calculation areas were calculated with the help of numerical CFD modeling tools with different *L* distance of the gum from the cut plane of the nozzles (Figure 5).

The type and size of computational domains were unified. In addition, to specify the nature of the flow of the mixture and its interaction with the gingiva, a cylindrical BOI (body of influence) object was used, which significantly increases the density of the computational grid in the grid region but does not affect the geometry of the objects of interest — the nozzle and the gum.

The gingiva in such a computational setting is modeled using a model of a porous 1-mm thick medium, occupying the entire area transversely with respect to the computational domain. The flow of a liquid-air mixture inside a porous medium is described by the Darcy’s empirical law [4, 5].

In this approach, the Reynolds number is a dimensionless quantity that describes the relationship between inertial forces with respect to viscous forces. It is calculated as a function of the pore size or the particle diameter (*d*_p_) that make up the porous medium:

Re=ρυSdpη,

where ρ is the density of the fluid; υ_s_ is the vector of the liquid surface velocity [6]; η is the coefficient of dynamic viscosity.

It follows from these conditions that the term of the momentum conservation equation with respect to the change in pressure in the physical region for a fluid flowing through isotropic porous medium is described as

ΔP=−η
υ→SK,

where η is the dynamic viscosity of the fluid; *K* is the permeability of the porous medium (m^2^); υ_s_ is the fluid surface velocity vector, which defines the porous medium as continuous and neglects the influence of the geometric features of the internal structure of the porous medium.

The permeability of a porous medium *K* (in m^2^) is calculated from the equation

K=ηqϕ3⋅LΔP⋅F,

where q^φ3^ is the fluid volumetric flow rate (debit) (m^3^/s); η is the dynamic viscosity of the fluid (Pa·s), Δ*P*=*Р*1–*Р*2 is differential pressure (Pa); *L* is the length of the porous medium sample (m); *F* is the filtration area (m^2^).

It should be noted that the Ansys Fluent package uses the value 1/*K*.

When solving the current problem, taking into account the lack of data on gingival permeability in open sources, the porosity value was taken as φ=0.1, and the reverse permeability 1/K was considered anisotropic, so that its value along the normal to the gingiva (Y axis) exceeded the corresponding values on the X, Z axes.

## Results of mathematical modeling

**Figure 6.**
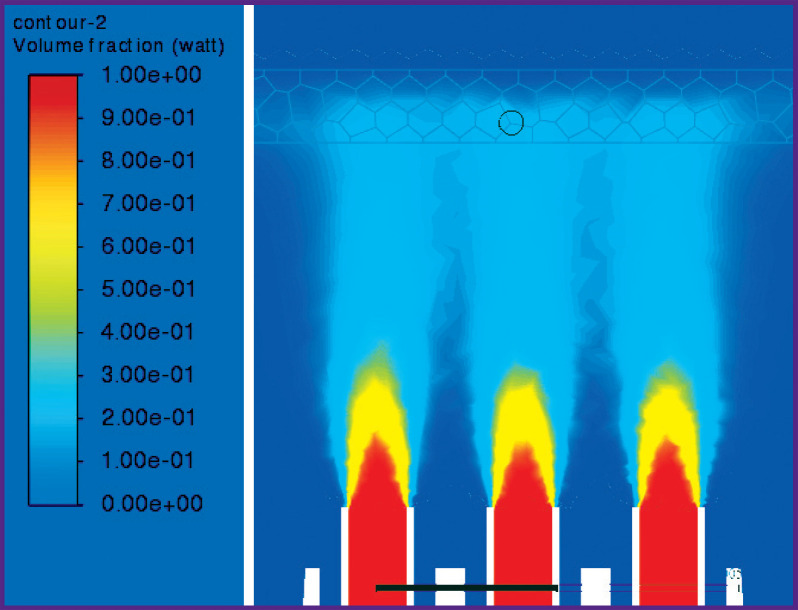
Liquid volume fraction distribution at *L*=5 mm

**Figure 7.**
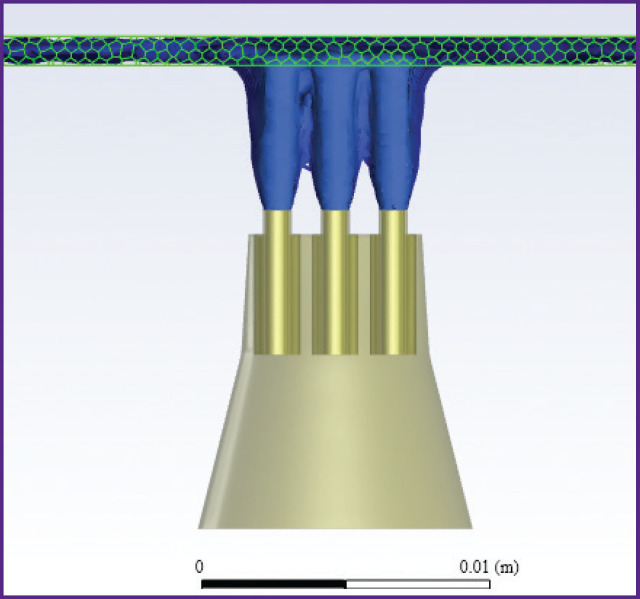
Distribution of isosurfaces of the liquid volume fraction in the range of 18–20% at *L*=5 mm

**Figure 8.**
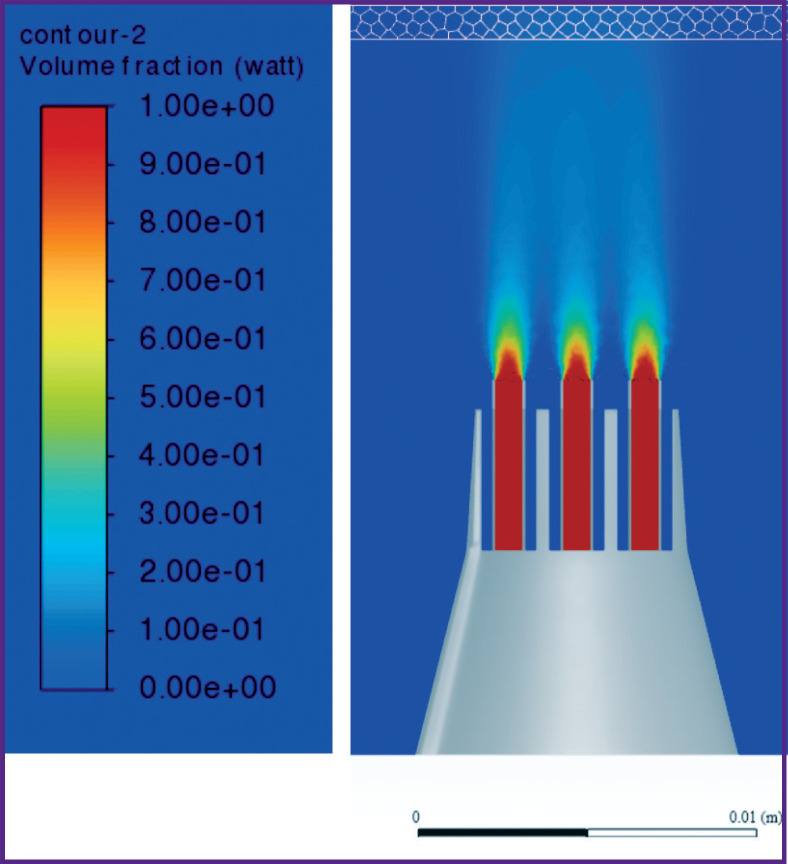
Liquid volume fraction distribution at *L*=10 mm

**Figure 9.**
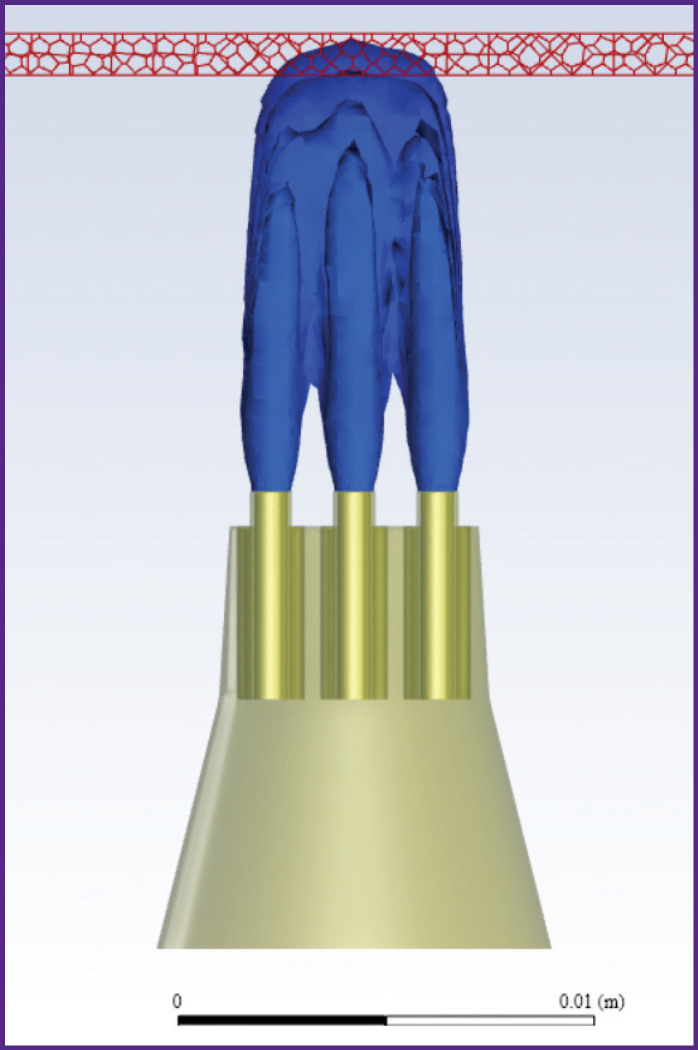
Distribution of isosurfaces of the liquid volume fraction in the range of 5–7% at *L*=10 mm

**Figure 10.**
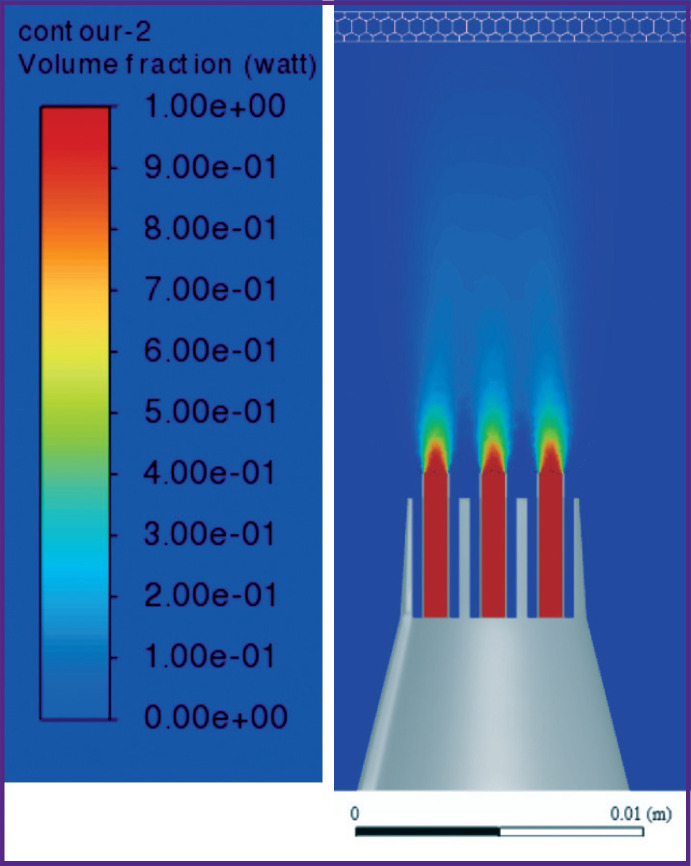
Liquid volume fraction distribution at *L*=15 mm

**Figure 11.**
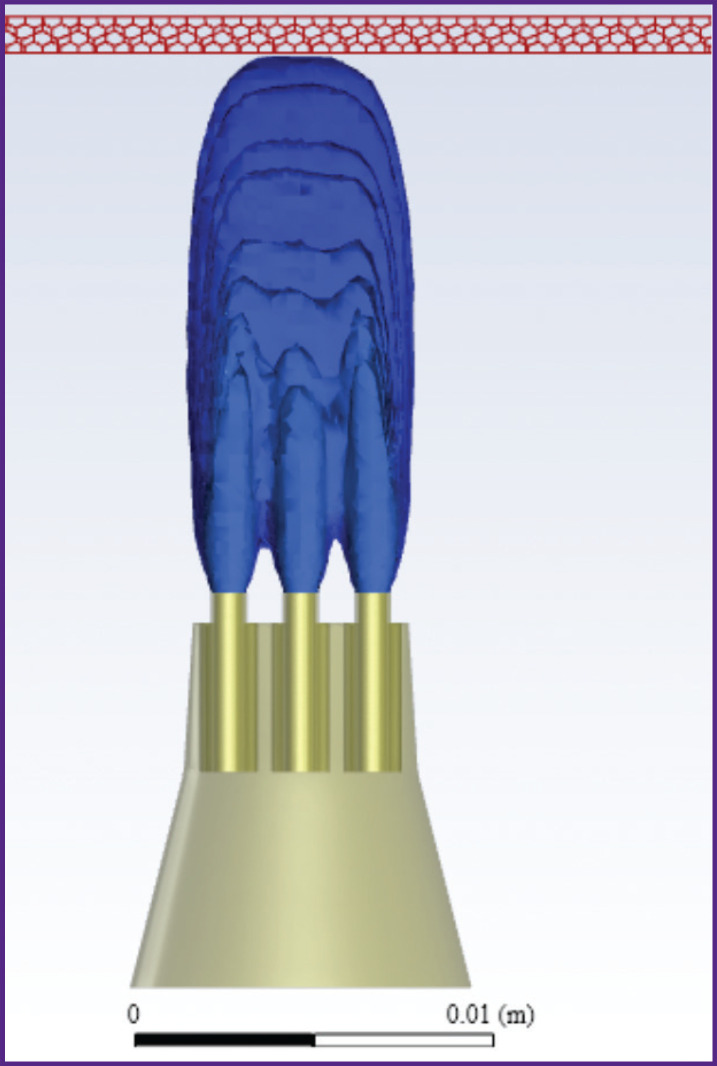
Distribution of isosurfaces of the liquid volume fraction in the range of 2–5% at *L*=15 mm

According to the results of the converged iterative process of solving the problem, the following results were obtained when the nozzle was alienated from the gum at distances *L*=5, 10, 15 mm (Figures 6–11).

Figures 6 and 7 show the contours of distribution of the liquid volume fraction in the mixture (the percentage of the liquid in the mixture relative to the sum of the components) at *L*=5 mm. At the same time, the liquid volume fraction close to the gum is distributed in the range of 18–20%. The mixture actually breaks through the gum, filling 0.8 mm of its thickness and spreading symmetrically to the sides up to 3 cm with the formation of a cavity. Thus, the distance of *L*=5 mm from the nozzles to the gums must be considered critical and traumatic, which correlates with medical practice.

Figures 8 and 9 show the distribution of the liquid volume fraction in the mixture at *L*=10 mm. Near the gingiva, the liquid volume fraction is distributed in a narrow range of 5–7%. The mixture touches the surface of the gums, slightly getting inside by 0.30–0.45 mm. Thus, the liquid is safely delivered inside the gums without injuring the patient.

Figure 10 shows the distribution of the liquid volume fraction in the mixture at the greatest distance of the nozzle from the gum surface (*L*=15 mm). In this case, the liquid volume fraction near the gum is distributed in the range of 2–5%. The mixture slightly touches the gum surface, getting inside at a distance of up to 0.2 mm, actually, exercising no effect on the gum, as shown in Figure 11.

Therefore, the developed mathematical model of the interaction of the liquid-air mixture with periodontal tissues when using the barophoresis technique in the clinical practice of periodontology indicates the effectiveness of its application for drug delivery to the gums. The optimal distance from the nozzle to the gum surface can be considered to be 10–15 mm. This distance is safe for the patient and allows the drug to be delivered to a depth of 0.45 mm.

## Conclusion

We have applied numerical modeling in solving the problem of finding the optimal distance from the nozzle to the gingival surface during barophoresis. The integrated approach that we used included the numerical solution of the Reynolds-averaged Navier–Stokes equations. The results have shown that the assumptions and coefficients adopted in setting the problem and solving it ensure the physicality of the results and indicate the feasibility of using barophoresis in medical practice.
